# TRPV4 Mechanotransduction in Fibrosis

**DOI:** 10.3390/cells10113053

**Published:** 2021-11-06

**Authors:** Ravi K. Adapala, Venkatesh Katari, Lakshminarayan Reddy Teegala, Sathwika Thodeti, Sailaja Paruchuri, Charles K. Thodeti

**Affiliations:** 1 Department of Physiology and Pharmacology, College of Medicine and Life Sciences, University of Toledo, Toledo, OH 43614, USA; Ravi.Adapala@Utoledo.edu (R.K.A.); Venkatesh.Katari@Utoledo.edu (V.K.); Lakshminarayan.Teegala@Utoledo.edu (L.R.T.); Sailaja.Paruchuri@Utoledo.edu (S.P.); 2 Northeast Ohio Medical University, Rootstown, OH 44272, USA; sthodeti@neomed.edu

**Keywords:** calcium, extracellular matrix, fibroblast, fibrosis, mechanotransduction, myofibroblast, TGF-β, TRPV4

## Abstract

Fibrosis is an irreversible, debilitating condition marked by the excessive production of extracellular matrix and tissue scarring that eventually results in organ failure and disease. Differentiation of fibroblasts to hypersecretory myofibroblasts is the key event in fibrosis. Although both soluble and mechanical factors are implicated in fibroblast differentiation, much of the focus is on TGF-β signaling, but to date, there are no specific drugs available for the treatment of fibrosis. In this review, we describe the role for TRPV4 mechanotransduction in cardiac and lung fibrosis, and we propose TRPV4 as an alternative therapeutic target for fibrosis.

## 1. Introduction

Fibrosis is characterized as the accumulation of excessive extracellular matrix (ECM) in response to injury or insult resulting in stiffening of the tissue. Physiologically, organs comprise cells that are enclosed within an acellular ECM. ECM varies depending on the function of an organ, which dictates its structure and mechanical properties [1,2]. ECM homeostasis is a tightly regulated process that works by balancing the synthesis and degradation of ECM. Imbalance in this process results in the excessive deposition of ECM and results in fibrosis. Fibrosis is an adaptive wound healing process that starts with the recruitment of inflammatory cells to secrete various cytokines and chemokines, such as transforming growth factor-beta (TGF-β), tumor necrosis factor-alpha (TNF-α), platelet-derived growth factor (PDGF), and interleukins. In response to injury, infiltrated leukocytes, as well as resident fibroblasts, degrade ECM by secreting matrix metalloproteases (MMP), which enables cell migration and creates a path for blood vessel growth [3]. Changes in the degradation of collagen due to injury or insult initiates increased production of ECM proteins and tissue inhibitors of metalloproteases (TIMPS) at the site of injury by differentiating fibroblasts into myofibroblasts (MF). Although the increased production of ECM proteins is beneficial initially, the prolonged accumulation of ECM disrupts their structural dynamics and cellular communication, resulting in poor function and fibrosis in vital organs that result in mortality and morbidity [1,3,4].

Current anti-fibrotic therapies are mainly focused on inhibiting the secretion of pro-inflammatory cytokines and their activity. However, emerging data suggests that both preventing and ameliorating ECM expansion from the overproduction of collagen are crucial steps in successfully identifying anti-fibrotic therapies [5]. The recent development on anti-fibrotic therapies showed the targeting of soluble factors and their signaling molecules such as TGF-β1, Angiotensin II, Endothelin-1, PDGF, and CCN2 [6]. Losartan, an antihypertensive angiotensin II type 1 receptor blocker, controls TGF-β1 availability and signaling in fibrin-1 mutation-induced Marfan syndrome [7,8]. Orally active pirfenidone inhibits collagen by reducing the activation of latent TGF-β by antagonizing the TGF-β activating convertase enzyme [9]. Moreover, plant alkaloid halofuginone inhibits collagen synthesis through TGF-β1 signaling by reducing smad3 phosphorylation [10,11]. Directly targeting TGF-β and its downstream signaling molecules is not a viable option to reduce fibrosis due to its regulation in other signaling mechanisms and its adverse effects such as liver dysfunction, restenosis, and cardiovascular effects [12]. Therefore, there is an urgent necessity for the development of alternative strategies that could repair fibrotic condition.

## 2. Fibroblast Differentiation and Signaling Mechanism

**Figure 1:**
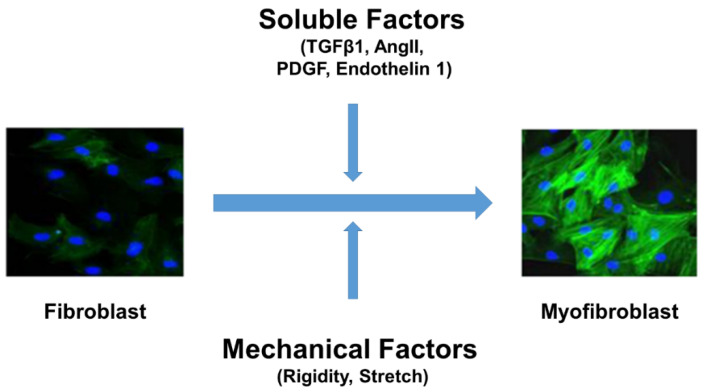
Fibroblast differentiation into myofibroblasts is a key event in fibrosis. Both soluble factors (TGF-β or Ang II or PDGF or Endothelin 1) and mechanical factors (stretch and stiffness) are required for cardiac fibroblasts differentiation into hyper-secretory and hyper-contractile phenotype myofibroblasts (α-SMA (green) expression and incorporation into stress fibers are known markers of myofibroblasts; nuclei were stained in blue). Ang II = angiotensin II; α-SMA = α-smooth muscle actin; TGF-β = transforming growth factor β. PDGF = Platelet derived growth factor.

**Figure 2:**
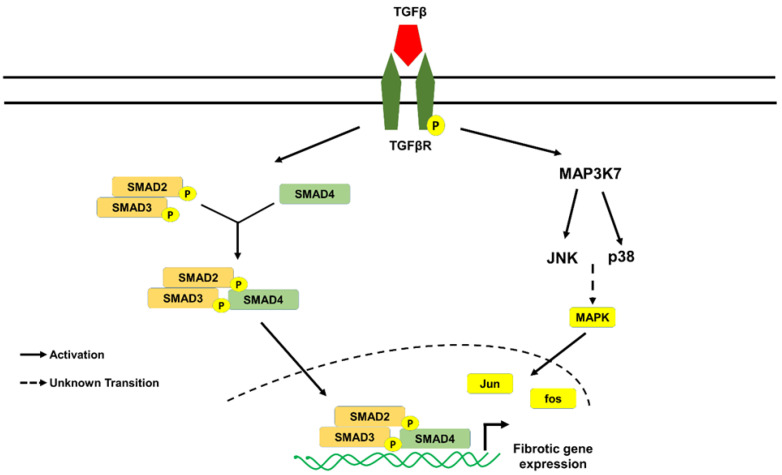
TGF-β signaling fibroblasts differentiation into myofibroblasts. TGF-β is the major soluble factor implicated in fibroblast differentiation. The canonical pathway includes binding of TGF-β to the TGF-β receptors resulting in the activation of SMAD2/3, which forms a complex with SMAD4, translocates to nucleus, and induces fibrotic gene expression. In contrast, non-canonical pathway includes p38 MAPK/JNK pathway, which activates Jun/Fos-dependent fibrotic gene expression. α-SMA = α-smooth muscle actin; TGF-β = transforming growth factor β; TGFβR = transforming growth factor β receptor.

Although various cells secrete ECM, fibroblasts are principal cells that maintain the homeostatic balance of ECM and preserve structural integrity of the tissue. Recently, single-cell RNA sequencing and cell lineage studies revealed that fibroblasts are heterogenous and that only less than 20% of fibroblast enriched gene overlap across heart, skeletal muscle, intestine, and bladder tissues [13]. Lineage tracing studies revealed that the origin of fibroblasts was from resident fibroblasts in pressure overload mice during development and fibrosis [14]. After injury or insult, fibroblasts differentiate into hyper-secretory and hyper-contractile phenotype called myofibroblasts. Soluble factors (TGF-β or Angiotensin II or PDGF or Endothelin 1) and mechanical forces (Stretch and/or ECM rigidity) play a vital role in the differentiation of fibroblasts into myofibroblasts (Figure 1) [4,15,16,17,18,19,20,21,22,23,24,25,26,27]. Most studies demonstrated that TGF-β and it’s signaling plays a central role in the initiation and progression of fibroblasts differentiation [28,29]. TGF-β acts through canonical SMAD signaling or noncanonical MAPK/JNK/p38 signaling pathways which are considered key regulators in fibrotic gene expression (Figure 2). Genetic deletion of MAPK2 reduces alpha-smooth muscle actin (α-SMA) expression in TGF-β1 stimulated fibroblasts. p38 is a key regulator in fibroblasts differentiation, as it plays a role in controlling both cytokine and mechanical signaling induced by α-SMA expression through the activation of serum response element1. Deletion of p38 reduces ischemia-induced fibrosis and myofibroblasts formation in vivo. Fibroblast-specific activation of p38 enhances myofibroblasts formation, resulting in perivascular and interstitial fibrosis in the heart, lung, and kidney [30]. In addition to soluble factor signaling, changes in ECM stiffness during pathological remodeling promotes fibroblast differentiation. Fibroblasts sense changes in mechanical properties through integrins and dictate actomyosin remodeling via modulating Rho-ROCK pathway. Studies have shown that integrin activation modulates phosphorylation of YAP and ILK, suggesting the role of the hippo pathway in fibroblast differentiation [31]. Furthermore, stiffening the ECM enhances Wnt signaling molecules through the activation of the integrin/focal adhesion kinase pathway. Recent evidence demonstrated that TGF-β signaling cross-talks with other pathways such as Wnt/β-catenin and YAP/TAZ pathway via upregulating MMP production and TGF-β1 in lung and cardiac fibrosis [31,32,33,34]. Although integrins sense and induce fibrotic gene expression in response to mechanical stress, the exact mechanisms and molecules involved in converting these signals into biochemical signals are not known.

In addition to these signaling mechanisms, calcium plays critical role in fibroblast differentiation by modulating various pathways related to proliferation, migration, fibrotic gene expression, and contraction [35,36]. Unlike other cells, calcium regulation in fibroblasts is not studied well. Intracellular regulation of calcium is dependent on both store operated and receptor operated mechanisms and lack of ryanodine receptor expression and activation in fibroblasts, suggesting that inositol triphosphate (IP3) calcium signaling mechanisms might play a role in fibroblast activation. Previous studies have shown that receptor operated calcium was enhanced in fibroblasts derived from Alzheimer disease compared to healthy fibroblasts [37]. Recently, Mukherjee et al. showed nifedipine reduced TGFβ1 induced calcium in human pulmonary fibroblasts and prevented bleomycin induced pulmonary fibrosis without affecting inflammation, suggesting the role of calcium in fibrosis [36,38]. Many G-protein coupled receptors have shown an increase in intracellular calcium through IP3 dependent pathways. For example, angiotensin II receptor AT1 activation increased intracellular calcium and is responsible for angiotensin II induced collagen production. Other G-protein coupled receptor activations by angiotensin II include bradykinin involved in IP3-dependent calcium regulation in rat cardiac fibroblasts [39]. Although these mechanisms have shown calcium mediated regulation of fibroblast differentiation, the role of TRP channels in fibroblast differentiation is not known until recently.

## 3. TRP Channels in Fibroblasts

**Figure 3:**
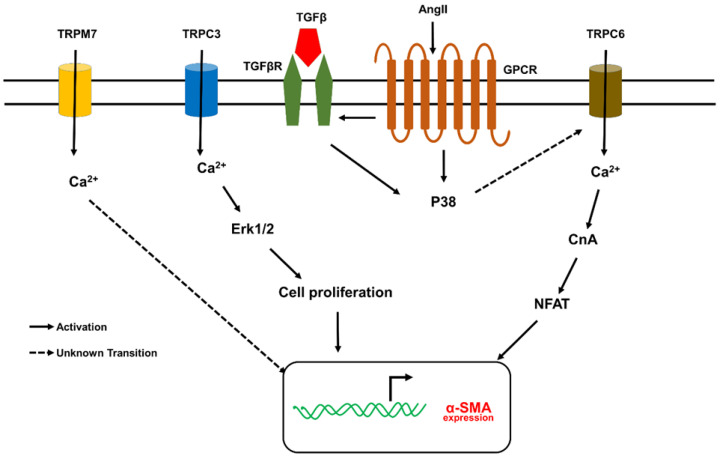
Schematic showing possible mechanisms by which TRP channels (other than TRPV4) regulate fibroblast differentiation. TRPC3, TRPC6, and TRPM7 have been implicated in fibroblast (atrial and ventricular) differentiation. TGF-β shown to influence the expression/activity of these three TRP channels. TRPC3 signaling demonstrated to be critical for atrial fibroblast proliferation, migration, and fibrosis. The possible signaling mechanism appears to be AngII/AT1R/TGF-β1 mediated increase in TRPC3 expression via NFAT dependent downregulation of miR-26, which in turn activates fibroblast proliferation through ERK1/2. TGF-β was also shown to increase the expression of TRPC6 channels via p38/SRF and then calcium influx from TRPC6 induces fibroblast differentiation through CnA/NFAT signaling. TRPM7 shown to regulate mechanical stretch-induced fibroblast differentiation (atrial and adventitial) though p38 MAPK/JNK pathway. Ang II, angiotensin II; CnA, calcineurin; miR-26, microRNA26; NFAT, Nuclear factor of activated T-cells; ROCK, Rho associated protein kinase; SRF, serum responsive factor; α-SMA, α-smooth muscle actin; TGF-β, transforming growth factor β.

Recently, transient receptor potential (TRP) channels have become one of the most important families of ion channels in fibrosis because of their activation in response to various biochemical and mechanical stimuli [36,40]. TRP channels are divided into six subfamilies, including TRPC (canonical), TRPV (vanilloid), TRPM (melastatin), TRPA (ankyrin), TRPP (polycystin), and TRPML (mucolipin). Among many TRP channels, TRPC1, TRPC2, TRPC3, TRPC5, TRPC6, TRPC7, TRPV2, TRPV4, and TRPM7 have been shown to be expressed in mouse, rat, and human cardiac fibroblasts (Figure 3). TGF-β1 was shown to upregulate TRPC6 expression by modulating the p38/SRF pathway, and TRPC6 dependent calcium promoted fibroblasts differentiation through the activation of the calcineurin/NFAT signaling pathway [41,42]. TGF-β1 induces TRPC3 activation via NFAT-mediated downregulation of miR-26 in atrial fibroblasts. TRPC3-mediated calcium influx further induced the proliferation and differentiation of fibroblasts through ERK1/2 [43]. Atrial fibroblasts isolated from AF patients exhibited increased TRPM7 expression, and inhibition of TRPM7 reduced TGF-β1 induced atrial fibroblast proliferation and differentiation. Although TRPM7 is expressed in ventricular fibroblasts, it is not involved in ventricular fibroblast differentiation [44,45]. There is limited work on the role of different TRP channels, except for TRPV4, in lung fibroblasts differentiation and lung fibrosis. Both TRPV1 and TRPA1 channel expression have been shown to increase in bleomycin-induced pulmonary fibrosis in guinea pigs [46]. However, samples from IPF patients showed no difference in the expression of TRPV1 and TRPA1 in airways compared to normal subjects [47]. Although many TRP channels are implicated in the disease process, TRPV4 gained prominence as a mechanosensor in cardiac and lung fibroblasts [20,21,22]. However, the role of TRPV4 mechanotransduction in fibrosis is not fully explored, and the current review discusses the mechanistic role of TRPV4 in cardiac and pulmonary fibrosis.

## 4. TRPV4 Structure and Mechanotransduction

TRPV4 is a non-selective cation channel that mediates calcium, magnesium, and sodium ions in response to biochemical and mechanical factors in many cell types including fibroblasts, endothelial cells, epithelial cells, and macrophages. TRPV4 channel consist of four monomers, and each monomer of TRPV4 is comprised of six transmembrane domains with a pore loop in between fifth and sixth transmembrane domains [48,49]. N-terminus and C terminus are located to the intracellular region and contain a variety of functional domains such as proline-rich, six ankyrin domains at N-terminus and TRP, MAP7 (microtubule-associated protein 7) binding domain, and calmodulin domain at C-terminus. Ankyrin domains and proline-rich domains interact with other proteins to regulate the downstream signaling process. A cytoskeletal protein PACSIN3 interacts with TRPV4 at the first proline-rich domain of NH2 terminus and mediates the response of TRPV4 to hypotonicity and warm temperature. Previous studies have shown that colocalization of TRPV4 with F-actin is required for hypertonic induced TRPV4 activation [48,49]. TRPV4 function has been well established in endothelial cells using calcium influx and patch clamp assays, but its functional expression in fibroblasts is still under studied. Previously, we and others have shown that TRPV4 activation induced rapid increase and decline of calcium influx in endothelial cells. In contrast, fibroblasts showed rapid increase but sustained calcium influx in response to TRPV4 agonists [20,21,23,24]. Patch-clamp recordings showed that TRPV4 agonists induced a typical large outward rectifier current at positive membrane potentials and an inward current at negative membrane potentials in both endothelial and fibroblasts [23,50,51,52]. Hatano et al. showed that TRPV4 activation by 4-α-PDD evoked non-selective cationic currents in rat cardiac fibroblasts, which were inhibited by ruthenium red [50]. Rahman et al. demonstrated evidence for TRPV4 current in human pulmonary fibroblasts with a large conductance change by TRPV4 agonists, 4-α-PDD, and GSK1016790A, and they are sensitive to the removal/inhibition of Na and Cl [51]. In fact, we found that GSK1016790A-induced a typical TRPV4 current with large outwardly rectifying current and small inward current in WT mouse cardiac fibroblasts (mCF), which is completely absent in TRPV4KO mCF (unpublished). In addition to agonist dependent TRPV4 activation studies, recently Sianatil et al. demonstrated that mechanical activation at the cell–substrate interface induced TRPV4 currents in HEK293 cells expressing TRPV4. Furthermore, they have shown that point mutation(s) at different sites of TRPV4 evoked different kinds of currents in response to mechanical activation at cell–substrate interfaces [53]. Mutations in TRPV4 cause a number of skeletal and peripheral diseases such as skeletal dysplasia, arthropathies, and neuropathies [54]. TRPV4 has been implicated as a mechanosensor towards mechanical forces such as osmotic swelling, mechanic stretch, shear stress, and ECM stiffness [20,21,23,55,56,57]. Therefore, it is plausible that the activation of TRPV4 increases the abundance of calcium through various triggers of biochemical and mechanical forces and dictates the fate of the cell.

**Figure 4:**
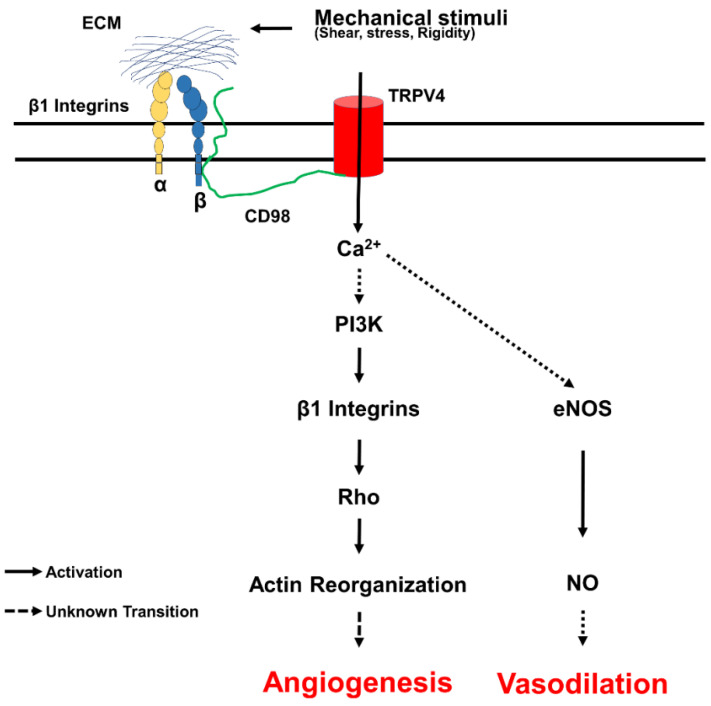
TRPV4 mechanotransduction in endothelial cells. Cells sense mechanical forces exerted on extracellular matrix through integrins in focal adhesion. Application of mechanical force through integrins results in ultra-rapid activation of TRPV4 channels via CD98 proteins. TRPV4 mediated calcium influx activates additional integrins through PI3 Kinase, which induces transient downregulation and later stabilization of Rho facilitating cytoskeletal reorientation and directed migration of EC required for physiological angiogenesis. TRPV4-mediated calcium influx can activate endothelial nitric oxide synthase (eNOS) and generation of nitric oxide (NO), which induces vasodilation.

TRPV4’s role in mechanotransduction was first identified in a screen for mutants defective in olfaction in *Caenorhabditis elegans* (*C. elegans*) such as Osm-9 gene (TRPV4 homologue), which plays an important role in various sensory functions such as chemosensation and osmosensation [58,59]. One of the first pieces of evidence for TRPV4 mechanosensitive role in mammalian cells comes from its activation in response to hypotonic cell swelling [20,55]. However, TRPV4 recently gained prominence as a mechanosensor in the endothelium. Kohler et al. were the first to demonstrate that TRPV4 is a mechanosensor of shear stress, and it is required for nitric oxide (NO) production and vasodilation in rat carotid arteries [60]. Using TRPV4KO mice, Kohler and colleagues later confirmed that TRPV4 indeed mediates flow-induced vasodilation in resistant arteries [61]. In search of the mechanosensor of cyclic stretch in endothelial cells, we found that TRPV4 is activated in response to cyclic stretch and that TRPV4 mediated calcium influx is required for cyclic strain-induced reorientation of endothelial cells [23]. Furthermore, by using magnetic pulling cytometry (MTC), we demonstrated that mechanical force application through β1 integrins via magnetic beads coated with either RGD peptide or 12G10 antibodies results in TRPV4-dependent ultra-rapid calcium influx (within 4 milliseconds) in endothelial cells [56]. We found that TRPV4 is localized to focal adhesions and is activated by mechanical force transfer through β1 integrin cytoplasmic tail binding protein, CD98 [56]. Deletion of a distal region of β1 integrin cytoplasmic domain that binds to CD98 or direct application of force to CD98 activated TRPV4 induced calcium signals within focal adhesions, confirming that TRPV4 is a mechanosensory in endothelial cells. We then dissected the downstream signaling mechanism and revealed that TRPV4 mediates cyclic strain-induced endothelial cell reorientation through integrin to integrin signaling via the activation of PI3K, which in turn regulates cytoskeletal reorientation and angiogenesis through the modulation of Rho (Figure 4). Shear stress also activates TRPV4, resulting in Ca^2+^ influx that stimulates other Ca^2+^ dependent IP3 receptors in endothelium vasodilation [62,63,64,65,66]. Shear force induces relocation of TRPV4 channels from adherens junctions and reduces the interaction of TRPV4 with ß-catenin that is controlled by FAK and α5β1 integrins [67]. Importantly, the critical role for TRPV4 mechanotransduction in controlling endothelial physiology is elucidated by the deletion or downregulation of TRPV4. We demonstrated that the absence or downregulation of TRPV4 imparts abnormal mechanosensitivity toward matrix rigidity in endothelial cells via the upregulation of Rho/Rho kinase activity, resulting in enhanced cell spreading, migration, and abnormal angiogenesis [57]. Furthermore, we found that the absence of TRPV4 increases endothelial proliferation via upregulating cyclin-ERK-pathway [68]. In a subcutaneously implanted syngeneic tumor model in vivo, TRPV4KO mice exhibited increased tumor growth, abnormal angiogenesis, vascular destabilization, and metastasis. Furthermore, the pharmacological activation of TRPV4 normalized abnormal vasculature and improved cancer therapy [57]. Pertinent to these findings, the endothelial-specific deletion of TRPV4 recapitulated similar tumor phenotypes in global TRPV4KO mice, and the molecular mechanism appears to be the knockdown of TRPV4 that increases translocation and phosphorylation of VEGFR2 through YAP, which is activated by the Rho/Rho kinase pathway [69,70]. Since TRPV4 can be activated by mechanical forces including cyclic strain, shear stress, and ECM stiffness, which then mediates actin remodeling, it is possible that TRPV4 could play a role in fibroblast differentiation and fibrosis.

## 5. TRPV4 Mechanotransduction in Cardiac Fibrosis

TRPV4 is expressed in all major cardiac cells (cardiomyocytes, fibroblasts, and endothelial cells) and is required for the physiological function of the heart [71,72,73] (Adapala, Unpublished). Using genome-wide analysis, Zhou et al. reported that TRPV4 might have a potential role in myocardial infarction [74]. However, Hatano et al. were the first to show the expression of TRPV4 in rat cardiac fibroblasts [50]. Since mechanical factors are critical for cardiac fibroblast differentiation and TRPV4 is a mechanosensitive ion channel in endothelial and other cells, we investigated the role of TRPV4 in the differentiation of cardiac fibroblasts into myofibroblasts. In order to determine this, we measured TRPV4 expression using RT-PCR and calcium imaging, and we found that rat cardiac fibroblasts (rCF) functionally express TRPV4 channels. Furthermore, we found that TGF-β1 induced robust differentiation of rCF to MF, as indicated by the incorporation of α-SMA into stress fibers, which is attenuated by the inhibition of TRPV4 with AB159908 or siRNA knockdown of TRPV4 [21]. Next, in order to explore whether TRPV4 regulates TGF-β1-induced rCF differentiation though mechanical signaling, we cultured rCF on ECM gels with varying stiffness (low-98 Pa; intermediate-370 Pa and high-2280 Pa). Indeed, we found that TGF-β1-induced differentiation of rCF cultured on high stiffness gels but not on low stiffness (98 Pa, 370 Pa) gels. Importantly, even saturated concentrations of TFG-β1 failed to induce rCF differentiation on low stiffness gels, confirming that soluble factors alone are insufficient and mechanical factors are required for fibroblast differentiation to myofibroblasts. Notably, pharmacological inhibition of TRPV4 significantly diminished TGF-β1-induced rCF differentiation on high stiffness gels, suggesting that TRPV4 is required for TGF-β-induced fibroblast differentiation. Furthermore, TGF-β1 stimulation also increased TRPV4 expression and function in rCF [21]. These findings, for the first time, demonstrated that TRPV4 mediates fibroblast differentiation by integrating soluble (TGF-β1) and mechanical (stiffness) signaling.

Although these studies have shown that TRPV4 promotes fibroblast differentiation, the mechanotransduction mechanism downstream of TRPV4 and its role in cardiac fibrosis are not known. In order to investigate this, cardiac fibroblasts (mCF) were first isolated from WT and TRPV4KO mice. While hypotonicity induced robust calcium influx in WT mCF, this response was significantly attenuated in TRPV4KO mCF, providing direct evidence that TRPV4 is a mechanosensor in mCF [20]. Furthermore, when cultured on ECM gels that mimic fibrotic hearts (8 and 50 kPa), TGF-β induced α-SMA expression and their incorporation into stress fibers in WT mCF but not in TRPV4KO mCF, confirming that TRPV4 mechanotransduction is required for mCF differentiation into MF. Furthermore, the inhibition of Rho kinase using Y27632 reduced TGF-β1 induced mCF differentiation, and TRPV4 antagonist AB155908 suppressed TGF-β1 induced Rho kinase activity, suggesting that the Rho kinase is downstream of TRPV4 in TGF-β1-induced CF differentiation. Furthermore, stimulation with TRPV4 agonist GSK1016790A or TGF-β1 increased nuclear translocation of myocardin related transcription factor-A (MRTF-A) in mCF, which was inhibited by TRPV4 antagonist, AB1559908. Finally, siRNA knockdown of MRTF-A reduced TRPV4, and TGF-β1 induced CF differentiation, suggesting TRPV4 regulates TGF-β1-induced CF differentiation by the modulating Rho/Rho kinase MRTF axis [20]. Other studies have demonstrated that TRPV4 mediated calcium influx is required for human CF differentiation via regulating the MAPK/ERK pathway; however, this is based on one single pharmacological inhibitor [75]. It was shown that TRPV4 upregulation in diabetes induced rat cardiac fibroblasts and pharmacological treatment with TRPV4 antagonist reduced cardiac fibrosis in streptozotocin induced diabetic rat model [76]. Although, these studies delineated the downstream signaling mechanisms of TRPV4 in TGF-β1 induced CF differentiation, TGF-β1-induced TRPV4 activation in CF is still elusive. Recently, we found that the inhibition of p38 MAPK reduced TGF-β1-induced TRPV4 membrane translocation in human cardiac fibroblasts (unpublished) suggesting that TGF- β may activate TRPV4 through the non-canonical pathway. Further studies are required in order to validate the regulation of TRPV4 by TGF-β1 in cardiac fibrosis; however, recent findings from lung fibroblasts provided evidence for the role of PI3K in the activation of TRPV4 by TGF-β.

The role of TRPV4 mechanotransduction in cardiac fibrosis was demonstrated by using global TRPV4KO mice [20]. In WT and TRPV4KO mice subjected to myocardial infarction (MI) by permanently ligating LAD, 2D echocardiography showed that the absence of TRPV4 preserved cardiac function in TRPV4KO mice compared to WT mice in the following 8 weeks after MI. Importantly, picrosirius red and Masson’s Trichrome staining revealed increased fibrosis at the infarct with a scar and remote zones in WT hearts exposed to MI, while TRPV4KO-MI hearts show reduced fibrosis at the infarct with no fibrosis in remote zones [20]. These findings suggest that TRPV4 is required for cardiac fibroblast differentiation and cardiac fibrosis in vivo and that deletion of TRPV4 preserves cardiac function and protects the heart by reducing cardiac fibrosis.

## 6. Role of TRPV4 Mechanotransduction in Pulmonary Fibrosis

**Figure 5:**
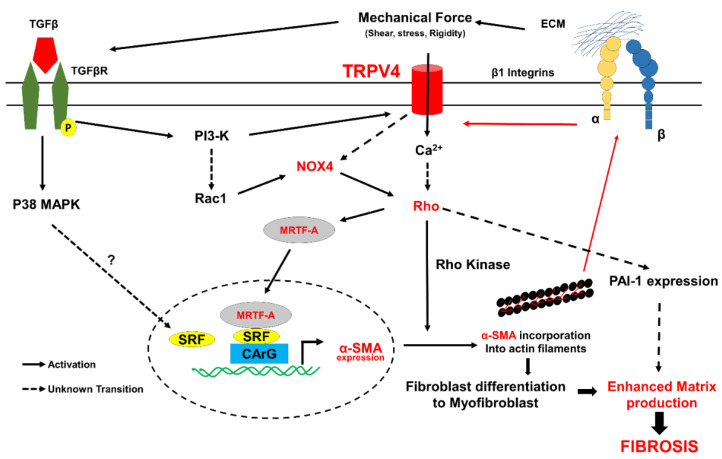
TRPV4 integrates soluble and mechanical signaling during fibroblast differentiation to myofibroblast. TRPV4 senses mechanical forces and activates Rho/Rho kinase pathway. Rho/Rho kinase dependent polymerization of actin releases MRTF-A from actin monomers, which then translocates into nucleus and induces α-SMA expression by binding with CArG sequences together with SRF. Rho/Rho kinase, on the other hand, enables α-SMA incorporation into the stress fibers, resulting in fibroblast differentiation into myofibroblasts. TGF-β activates PI3K-gamma, which forms complex with TRPV4; the complex is translocated to the plasma membrane and activates calcium influx at the Rho/MRTF-A pathway. TRPV4/TGF-β was also shown to activate NOX4 via PI3K/Rac1, which feeds into Rho pathway. In addition to inducing fibroblast differentiation to myofibroblasts and fibrotic gene expression, Rho/Rho kinase pathway activates PAI-1 expression and inhibits matrix degradation. Myofibroblasts are hypercontractile and apply tensional forces on the ECM and activate integrins, resulting in the release of TGF-β from latent complex inducing a positive feedback loop and enhancing fibroblast differentiation to myofibroblasts, excessive ECM production, and eventually fibrosis. Since, TRPV4 integrates soluble (TGF-β) and mechanical (integrin/Rho) signaling required for fibroblast differentiation and fibrosis, we propose TRPV4 as the novel therapeutic target for fibrosis.

Pulmonary fibrosis (PF) is a form of progressive lung disease that belongs to a large family of lung diseases called interstitial lung diseases. The most common type of PF is idiopathic pulmonary fibrosis (IPF), and its cause is unknown. Idiopathic pulmonary fibrosis (IPF) is a chronic, progressive, and devastating disorder characterized by the aberrant deposition of ECM resulting in pathological remodeling in the lung [77]. Although TRPV4 has been demonstrated to be expressed in lung epithelial, endothelial, and smooth muscle cells [78,79,80,81], the role of TRPV4 in pulmonary fibroblasts and fibrosis was first demonstrated following its role in cardiac fibroblasts [22]. TRPV4 expression was demonstrated to be upregulated in bleomycin-induced fibrosis in mice and that TRPV4-deficient mice were protected from bleomycin-induced pulmonary fibrosis via reducing fibroblast differentiation, resulting in reduced mortality rates. Consistently, inhibiting TRPV4 expression contributed to the abrogation of myofibroblast differentiation, which was recovered by TRPV4 reintroduction [22]. Interestingly, similarly to cardiac fibroblast differentiation, TRPV4-induced calcium influx is required for TGF-β1-induced lung myofibroblast differentiation. The molecular mechanism underlying TRPV4-mediated TGF-β1-induced lung fibroblast differentiation was demonstrated to be through a non-canonical SMAD-independent pathway involving actomyosin remodeling and increased nuclear translocation of MTRF-A. In separate studies, Paruchuri and colleagues demonstrated that TRPV4 mediates airway remodeling and fibrosis in a mouse model of allergen-induced asthma [24]. Importantly, fibroblasts isolated from patients with asthma exhibited enhanced TRPV4 expression and increased fibrotic gene expression (α-SMA) in response to TGF-β1 compared to normal fibroblasts. Mechanistically, they showed that TGF-β induced TRPV4 activation through phosphoinositide 3-kinase-alpha (PI3Kα), which is required for lung fibroblast differentiation. Importantly, this study also demonstrated that TRPV4 mediates *D. farina*-induced pulmonary inflammation and fibrosis through the activation of the TGF-β/PI3K/TRPV4/Rho pathway. The activated Rho pathway in turn activates p38 MAPK-dependent fibrotic gene expression, which results in enhanced collagen and fibronectin production and the simultaneous activation of plasminogen activator inhibitor 1 (PAI-1) resulting in reduced matrix degradation. These findings highlight the regulated role of TRPV4 on p38 and PAI-1 in lung fibrosis [24]. In contrast to PI3Kα’s role in pulmonary fibrosis, Grove et al. reported that TRPV4 promotes trans-differentiation of human and mouse lung myofibroblasts through interaction with PI3Kγ, forming intracellular TRPV4-PI3Kγ complexes [82]. TGF-β was shown to induce the recruitment of TRPV4-PI3Kγ complexes onto the plasma membrane and increased the activities of both TRPV4 and PI3Kγ. They showed that TRPV4 and PI3Kγ proteins are required for myofibroblast trans-differentiation and observed increased production of α-SMA and its incorporation into stress fibers, cytoskeletal changes, collagen I production, and contractile force [82]. Although the exact reason for the requirement of different PI3K isoforms in these studies is not clear, it could be due to different fibroblast cells used. Furthermore, recent studies from Paruchuri and colleagues demonstrated a critical role for NOX4 and reactive oxygen species (ROS) in TRPV4-mediated TGF-β1 induced lung fibroblast differentiation and *D. farina*-induced airway remodeling [83]. Specifically, NOX4 was shown to regulate TGF-β1-induced fibroblast differentiation via the activation of MRTF-A and increased PAI-1 expression, suggesting that NOX4 regulates fibrotic gene expression and matrix remodeling. Interestingly, the inhibition of NOX4 activity did not change TRPV4 activity, suggesting that NOX4 is downstream of TRPV4 [83]. Fibroblast differentiation can also be modulated by nitric oxide (NO) [84,85]. Recently, Park et al. demonstrated that nitric oxide attenuated transforming growth factor-β-induced myofibroblast differentiation of human keratocytes [86]. Interestingly, it was demonstrated that NOX4 and generated hydrogen peroxide (H_2_O_2_) crosstalk with NO pathway increased myofibroblast differentiation [87]. Although TRPV4 activation promotes NO production through the activation of nitric synthase in endothelial cells [88,89], its role in nitric oxide production in fibroblasts is not known. Taken together, these findings suggest that TRPV4 regulates TGF-β1/mechanical force-induced fibroblasts differentiation through Rho/NOX4/MRTF-A (Figure 5) signaling molecules and identifies TRPV4 as a potential novel therapeutic target for regulating cardiac and pulmonary fibrosis.

## 7. Summary and Clinical Implications

Although fibrosis has long been considered as the basis for many diseases, there are no specific efficient drugs available in the market. A few drugs such as dapagliflozin, Ertugliflozin, Metoprolol succinate, and Enalapril are still at phase III/IV clinical trials to treat fibrosis, and molecular mechanisms are yet to be known (https://clinicaltrials.gov; accessed on 18 October 2021; Supplementary Tables S1 and S2). One of the reasons for this could be that the research is focused on targeting soluble factors despite of our knowledge that mechanical factors are equally important for fibroblast differentiation. Moreover, the majority of reports on fibroblast differentiation are focused on fibrotic gene expression such α-SMA as the end point. However, α-SMA incorporation into the stress fibers is one of the hallmarks of myofibroblasts. Integrins, FAK, and other cytoskeletal proteins have been implicated as mechanical factors in fibroblast differentiation, but the upstream regulator is not known until now. Furthermore, targeting integrin could have off-target effects as integrin mediated cell adhesion is critical for the survival and proliferation of almost all cells in the body. We think TRPV4 is an ideal candidate as it was shown to integrate soluble and mechanical signaling during fibroblast differentiation to myofibroblasts (Figure 5). TRPV4 mechanotransduction is unique in the sense that it is not only involved in α-SMA expression but also in its incorporation into stress fibers. We speculate that the later process increases myofibroblast contraction and tensional forces on ECM molecules inducing the formation of matured fibers and increasing ECM stiffness. These tensional forces could then activate additional integrins, which were shown to release TGF-β from latent-TGF-β complexes that triggered a mechanotransduction positive feedback loop and inducing fibroblast differentiation. In addition to activating TGF-β-mediated pathways, TRPV4 mechanotransduction also modulates ECM remodeling by increasing the expression of PAI-1 (Figure 5). Therefore, it is plausible that targeting TRPV4 could inhibit fibrosis by inhibiting multiple pathways. Genetic deletion of TRPV4 was demonstrated to be protective against MI-induced cardiac fibrosis, bleomycin, and *D. farina*-induced lung fibrosis, supporting such a possibility. Interestingly, the absence of TRPV4 did not affect infarct size or scar but did reduce remote zone fibrosis in hearts subjected to MI, suggesting that targeting TRPV4 may inhibit unwanted/excessive fibrosis. Although current knowledge on TRPV4 role in fibrosis is limited to the global knockout of TRPV4, in vitro studies support a role for fibroblast TRPV4 in their differentiation and fibrosis. Nevertheless, further studies with fibroblast specific TRPV4KO mice are needed in order to unequivocally confirm the role of fibroblast TRPV4 mechanotransduction in fibrosis. TRPV4 selective inhibitors have so far shown no known side effects and appear to be cardioprotective, indicating that TRPV4 could be a viable specific target for fibrosis.

## Supplementary Materials

The following are available online at https://www.mdpi.com/article/10.3390/cells10113053/s1, Table S1: Drugs currently used in clinical trials for the treatment of Cardiac fibrosis (adapted from https://clinicaltrials.gov; accessed on 18 October 2021), Table S2: Drugs in clinical trials for the treatment of Idiopathic Pulmonary fibrosis (adapted from https://clinicaltrials.gov; accessed on 18 October 2021).
