# Supplementary material for: TRPV4 Mechanotransduction in Fibrosis
Source: Cells. 2021 Nov 6;10(11):3053. doi: 10.3390/cells10113053 (PMC8619244; doi:10.3390/cells10113053)
Supplement: Supplementary file 1 [file cells-10-03053-s001.zip › cells-1394121-supplementary.pdf]

Supplementary Table S1: Drugs currently using in clinical trials for the treatment of Cardiac fibrosis (adapted from https://clinicaltrials.gov)

S. No	NCT Number	Title	Conditions	Interventions	Mode of Action	Phases	# patients Enrolled	Sponsor/Collaborators
1	NCT02932566	The Efficacy and Safety of Pirfenidone in Patients With Heart Failure and Preserved Left Ventricular Ejection Fraction	Cardiac Failure	Drug: Pirfenidone<br>Drug: Placebo	Inhibits TGF-β1	Phase 2	129	Manchester University NHS Foundation Trust<br>National Institute for Health Research, United Kingdom<br>University of Manchester<br>University of Liverpool, Clinical Trials Research Centre<br>Hoffmann-La Roche
2	NCT02432885	Myocardial Fibrosis Progression in Duchenne and Becker Muscular Dystrophy - ACE Inhibitor Therapy Trial	Myocardial Fibrosis<br>Muscular Dystrophies	Drug: Enalapril	Ace Inhibitor	Phase 3	76	InCor Heart Institute, Federal University of Minas Gerais, University of Sao Paulo
3	NCT03991910	The Effect of Ramipril in Suppressing ST2 Expression in Rheumatic Mitral Stenosis Patients	Fibrosis; Heart<br>ACE Inhibitor	Drug: Ramipril 5Mg Oral Capsule<br>Drug: Placebo	Ace Inhibitor	Phase 3	66	Indonesia University
4	NCT03782259	Effects of SGLT-2 Inhibition on Myocardial Fibrosis and Inflammation as Assessed by Cardiac MRI in Patients With DM2	Myocardial Fibrosis<br>Myocardial Inflammation	Drug: dapagliflozin<br>Other: Placebo	SGLT-2 Inhibitor	Phase 4	60	University of Washington AstraZeneca
5	NCT02589977	Myocardial Perfusion, Oxidative Metabolism, and Fibrosis in HFpEF	Heart Failure, Diastolic<br>Diastolic Heart Failure<br>Hypertension	Drug: Regadenoson	A2A Adenosine receptor agonist	Phase 4	55	Marvin W. Kronenberg, M.D. Astellas Pharma US, Inc. Vanderbilt University Medical Center
6	NCT00663195	Effects of Spironolactone on Matrix Metalloproteinases (MMPs) in Heart Failure	Chronic Stable Heart Failure	Drug: spironolactone + furosemide	Diuretic	Phase 4	16	Tottori University Hospital
7	NCT04490681	Validation of ERtugliflozin for Inhibiting Cardiac Fibrosis Using Cardiac MRI and Laboratory Parameters in Korean Heart Failure Patients With Nonischemic Cardiomyopathy (VERTICAL)	Heart Failure With Non ischemic Cardiomyopathy	Drug: Ertugliflozin<br>Drug: Placebo	SGLT2 Inhibitor	Phase 3	52	Yonsei University
8	NCT01676285	Metoprolol Succinate in Cardiac Remodeling Related to Cirrhosis	Cirrhosis<br>Cirrhotic Cardiomyopathy<br>Cardiac Remodeling	Drug: Metoprolol succinate<br>Drug: placebo	Beta Blocker	Phase 3	125	University of Sao Paulo Fundação de Amparo à Pesquisa do Estado de São Paulo
9	NCT04128891	Study of Sacubitril/Valsaran on Myocardial Oxygenation and Fibrosis in Heart Failure With Preserved Ejection Fraction	Heart Failure With Preserved Ejection Fraction	Drug: Sacubitril-Valsartan	Neprylysin and Ace inhibitor	Phase 3	0	Flinders University
10	NCT02497937	A Study to Evaluate the Effect of the Transient Receptor Potential Vanilloid 4 (TRPV4) Channel Blocker, GSK2798745, on Pulmonary Gas Transfer and Respiration in Patients With Congestive Heart Failure	Heart Failure	Drug: GSK2798745<br>Drug: Placebo	TRPV4 Inhibitor	Phase 2	11	GlaxoSmithKline, Rochester, Minnesota, United States
12	NCT04704050	Effect of Dronedarone on Atrial Fibrosis Progression and Atrial Fibrillation Recurrence	Atrial Fibrillation<br>Atrial Fibrillation Recurrent	Drug: dronedarone 400 mg Oral Tablet<br>Drug: Placebo	Blocks potassium, sodium and Calcium channel blocker	Phase 4	330	Tulane University School of Medicine<br>University of Washington Marrek, INC<br>Cardiac Designs Inc. Sanofi Preventice<br>Tulane University

Supplementary Table S2: Drugs in clinical trials for the treatment of Idiopathic Pulmonary fibrosis (adapted from https://clinicaltrials.gov)

S.No	NCT Number	Title	Conditions	Interventions	Mode of Action	Phases	Enrollment	Sponsor/Collaborators
1	NCT01346930	Safety and Tolerability Study of Macitentan in Patients With Idiopathic Pulmonary Fibrosis	Idiopathic Pulmonary Fibrosis	Drug: Macitentan	Endothelin receptor antagonist	Phase 2	0	Actelion
2	NCT04598919	Saracatinib in the Treatment of Idiopathic Pulmonary Fibrosis	Idiopathic Pulmonary Fibrosis (IPF)	Drug: Saracatinab<br>Drug: Placebo	Src Inhibitor	Phase 1|Phase 2	100	National Jewish Health<br>Yale University<br>Icahn School of Medicine at Mount Sinai<br>AstraZeneca<br>National Center for Advancing Translational Science (NCATS)
3	NCT01504334	Safety and Efficacy Study of Pirfenidone to Treat Idiopathic Pulmonary Fibrosis (IPF)	Idiopathic Pulmonary Fibrosis	Drug: Pirfenidone<br>Drug: Placebo	Inhibits TGFβ	Phase 2	80	Beijing Kawin Technology Share-Holding Co., Ltd.
4	NCT00189176	Safety Study of Tetra thiomolybdate in Patients with Idiopathic Pulmonary Fibrosis	Idiopathic Pulmonary Fibrosis	Drug: Tetrathiomolybdate	Anti-copper agent	Phase 1|Phase 2	23	University of Michigan Coalition for Pulmonary Fibrosis
5	NCT03069989	Single Doses of GSK3008348 in Idiopathic Pulmonary Fibrosis (IPF) Participants Using Positron Emission Tomography (PET) Imaging	Idiopathic Pulmonary Fibrosis	Drug: GSK3008348<br>Drug: Placebo<br>Drug: [18F]-FBA-A20FMDV2	αvβ6 inhibitor	Phase 1	8	GlaxoSmithKline
6	NCT03727802	Safety, Tolerability, and Pharmacokinetic Study of TRK-250 for Patients With Idiopathic Pulmonary Fibrosis	Idiopathic Pulmonary Fibrosis	Drug: TRK-250<br>Drug: Placebo	Inhibits TGFβ	Phase 1	34	Toray Industries, Inc
7	NCT02168530	A Study of Oral Vismodegib for the Treatment of Idiopathic Pulmonary Fibrosis (IPF)	Idiopathic Pulmonary Fibrosis	Drug: placebo<br>Drug: vismodegib	Hedgehog pathway inhibitor	Phase 2	0	Hoffmann-La Roche
8	NCT01529853	To Evaluate the Effect of Different Doses of SAR156597 Given to Patients With Idiopathic Pulmonary Fibrosis (IPF)	Idiopathic Pulmonary Fibrosis	Drug: SAR156597<br>Drug: Placebo (for SAR156597)	Neutralizes free IL4 and IL13	Phase 1|Phase 2	24	Sanofi
9	NCT01725139	A Proof of Mechanism Study With GSK2126458 in Patients With Idiopathic Pulmonary Fibrosis (IPF)	Idiopathic Pulmonary Fibrosis	Drug: GSK2126458<br>Drug: Placebo	Phosphatidylinositol-3-Kinase Inhibitor	Phase 1	17	GlaxoSmithKline
10	NCT01135199	Pomalidomide for Cough in Patients With Idiopathic Pulmonary Fibrosis	Pulmonary Fibrosis	Drug: pomalidomide (CC-4047)	Inhibits angiogenesis	Phase 2	0	Stanford University Celgene Corporation
11	NCT04965298	Treating People With Idiopathic Pulmonary Fibrosis With the Addition of Lansoprazole	Idiopathic Pulmonary Fibrosis	Drug: Lansoprazole<br>Other: Matched placebo	Proton-pump inhibitor	Phase 3	298	Norfolk and Norwich University Hospitals NHS Foundation Trust Norwich Clinical Trials Unit
12	NCT00075998	The INSPIRE Trial: A Study of Interferon	Idiopathic Pulmonary Fibrosis	Drug: Interferon gamma-1b ("Actimmune")	IFN-gamma 1b	Phase 3	826	Inter Mune

		Gamma-1b for Idiopathic Pulmonary Fibrosis (IPF)	Lung Disease<br>Pulmonary Fibrosis					
13	NCT04429516	Morphine Sulfate/Placebo for the Treatment of PulmonAry Fibrosis Cough	Idiopathic Pulmonary Fibrosis	Drug: Morphine Sulfate<br>Drug: Placebo oral tablet	opioid receptors blocker	Phase 3	44	Royal Brompton & Harefield NHS Foundation Trust
14	NCT02874989	Targeting Pro-Inflammatory Cells in Idiopathic Pulmonary Fibrosis: a Human Trial	Idiopathic Pulmonary Fibrosis (IPF)	Drug: Dasatinib + Quercetin<br>Drug: Placebo	Reduces Senescence	Phase 1	26	Wake Forest University Health Sciences<br>Mayo Clinic<br>The University of Texas Health Science Center at San Antonio
15	NCT03650075	To Determine Safety and Tolerability of MG-S-2525 and to Evaluate Its PK Profile in Healthy Volunteers	Idiopathic Pulmonary Fibrosis (IPF)	Drug: MG-S-2525	Inhibits NOX4 and NOX1	Phase 1	81	Metagone Biotech Inc.
16	NCT04233814	Safety, Tolerability and Pharmacokinetic Study of LTI-03 in Healthy Adult Subjects	Idiopathic Pulmonary Fibrosis	Drug: Caveolin-1-Scaffolding-Protein-Derived Peptide (LTI-03)<br>Drug: Placebo	Caveolin	Phase 1	56	Lung Therapeutics, Inc
17	NCT00071461	Efficacy and Safety of Oral Bosentan in Patients With Idiopathic Pulmonary Fibrosis	Idiopathic Pulmonary Fibrosis	Drug: bosentan<br>Drug: Placebo	Endothelin receptor antagonist	Phase 2|Phase 3	158	Actelion
18	NCT00879229	ARTEMIS-PH - Study of Ambrisentan in Subjects With Pulmonary Hypertension Associated With Idiopathic Pulmonary Fibrosis	Idiopathic Pulmonary Fibrosis<br>Pulmonary Hypertension	Drug: Ambrisentan<br>Drug: Placebo	type A endothelin receptor antagonist)	Phase 3	40	Gilead Sciences
19	NCT01262001	Safety, Tolerability, and Efficacy Study of Idiopathic Pulmonary Fibrosis	Idiopathic Pulmonary Fibrosis	Drug: FG-3019	Anti-CTGF antibody	Phase 2	90	FibroGen
20	NCT00705133	Treprostinil Therapy For Patients With Interstitial Lung Disease And Severe Pulmonary Arterial Hypertension	Pulmonary Arterial Hypertension<br>Interstitial Lung Disease<br>Idiopathic Pulmonary Fibrosis	Drug: Treprostinil	Prostacyclin analog	Phase 2	15	Rajan Saggar United Therapeutics University of California, Los Angeles
21	NCT00903331	Macitentan Use in an Idiopathic Pulmonary Fibrosis Clinical Study	Idiopathic Pulmonary Fibrosis	Drug: ACT-064992 (macitentan)<br>Drug: Placebo	Endothelin receptor antagonist	Phase 2	178	Actelion
22	NCT04396756	Evaluation of Efficacy and Safety of PLN-74809 in Patients With Idiopathic Pulmonary Fibrosis	Idiopathic Pulmonary Fibrosis	Drug: PLN-74809<br>Drug: Placebo	αvβ6 and αvβ1 inhibitor	Phase 2	84	Pliant Therapeutics, Inc.
23	NCT04968574	A Study Evaluating the Safety and Efficacy of ENV-101 in Subjects With Idiopathic Pulmonary Fibrosis (IPF)	Idiopathic Pulmonary Fibrosis	Drug: taladegib<br>Drug: placebo	Hedgehog pathway inhibitor	Phase 2	60	Endeavor Biomedicines, Inc.
24	NCT03573505	An Efficacy and Safety Study of BG00011 in Participants With Idiopathic Pulmonary Fibrosis	Idiopathic Pulmonary Fibrosis	Drug: BG00011<br>Drug: Placebo	Blocks TGFβ	Phase 2	109	Biogen

25	NCT04419558	Zephyrus II: Efficacy and Safety Study of Pamrevlumab in Participants With Idiopathic Pulmonary Fibrosis (IPF)	Idiopathic Pulmonary Fibrosis	Drug: Pamrevlumab<br>Drug: Placebo	Anti-CTGF antibody	Phase 3	340	FibroGen
26	NCT01362231	A Study to Evaluate the Safety and Efficacy of GS-6624 (Formerly AB0024) in Patients With Idiopathic Pulmonary Fibrosis	Idiopathic Pulmonary Fibrosis	Drug: GS-6624	Inhibits cross linking of collagens by binding to LOXL2	Phase 1	48	Gilead Sciences
27	NCT03538301	JUNIPER: A Phase 2 Study to Evaluate the Safety, Biological Activity, and PK of ND-L02-s0201 in Subjects With IPF	Idiopathic Pulmonary Fibrosis	Drug: ND-L02-s0201<br>Other: Other: Placebo	Inhibits Heat shock protein 47	Phase 2	120	Nitto Denko Corporation
28	NCT03717012	Study of Pulmonary Rehabilitation in Patients With Idiopathic Pulmonary Fibrosis (IPF)	Idiopathic Pulmonary Fibrosis	Drug: Nintedanib<br>Other: Pulmonary rehabilitation program	Inhibits PDGF and, FGF receptor	Phase 4	19	Boehringer Ingelheim
29	NCT00287716	Three-Arm Study of the Safety and Efficacy of Pirfenidone in Patients With Idiopathic Pulmonary Fibrosis	Idiopathic Pulmonary Fibrosis	Drug: Pirfenidone<br>Drug: Placebo	Inhibits TGFβ	Phase 3	435	Genentech, Inc.
30	NCT03725852	A Clinical Study to Test How Effective and Safe GLPG1205 is for Patients With Idiopathic Pulmonary Fibrosis (IPF)	Idiopathic Pulmonary Fibrosis	Drug: GLPG1205<br>Drug: Placebo	G-protein Coupled receptor 84 Inhibitor	Phase 2	69	Galapagos NV
31	NCT01199887	Trial Of IW001 in Patients With Idiopathic Pulmonary Fibrosis	Idiopathic Pulmonary Fibrosis	Drug: IW001	Immunosuppression against Collagen V	Phase 1	30	Immune Works
32	NCT03928847	Fibroblast Specific Inhibition of LOXL2 and TGFbeta1 Signaling in Patients With Pulmonary Fibrosis.	Idiopathic Pulmonary Fibrosis	Drug: Epigallocatechin-3-gallate (EGCG)	Inhibits LOXL2 and TGFβ	Early Phase 1	35	University of California, San Francisco<br>National Heart, Lung, and Blood Institute (NHLBI)
33	NCT00879879	Losartan in Treating Patients With Idiopathic Pulmonary Fibrosis	Precancerous Condition	Drug: losartan	Angiotensin receptor blocker	Not Applicable	20	University of South Florida<br>National Cancer Institute (NCI)
34	NCT03830125	Safety, Tolerability, Pharmacokinetics, and Pharmacodynamics of BBT-877 in Healthy Subjects	Idiopathic Pulmonary Fibrosis	Drug: BBT-877, Single dose<br>Drug: Placebo group<br>Drug: BBT-877, Multiple doses	Autotaxin Inhibitor	Phase 1	88	Bridge Biotherapeutics, Inc.<br>KCRN Research, LLC
35	NCT00125385	Study of GC1008 in Patients With Idiopathic Pulmonary Fibrosis (IPF)	Idiopathic Pulmonary Fibrosis	Biological: GC1008	Neutralizes TGFβ isoforms	Phase 1	25	Genzyme, a Sanofi Company<br>Sanofi
